# Black Soldier Fly Larvae Influence Internal and Substrate Bacterial Community Composition Depending on Substrate Type and Larval Density

**DOI:** 10.1128/aem.00084-22

**Published:** 2022-05-09

**Authors:** Stijn J. J. Schreven, Hugo de Vries, Gerben D. A. Hermes, Giacomo Zeni, Hauke Smidt, Marcel Dicke, Joop J. A. van Loon

**Affiliations:** a Laboratory of Entomology, Plant Sciences Group, Wageningen University & Research, Wageningen, the Netherlands; b Laboratory of Microbiology, Agrotechnology & Food Sciences Group, Wageningen University & Research, Wageningen, the Netherlands; University of Queensland

**Keywords:** 16S rRNA gene, amplicon sequencing, *Hermetia illucens*, larval density, pH, manure, *Camelina sativa*, microbiota

## Abstract

Saprophagous fly larvae interact with a rich community of bacteria in decomposing organic matter. Larvae of some species, such as the black soldier fly, can process a wide range of organic residual streams into edible insect biomass and thus produce protein as a sustainable component of livestock feed. The microbiological safety of the insects and substrates remains a point of concern. Substrate-associated bacteria can dominate the larval gut microbiota, but the larvae can also alter the bacterial community in the substrate. However, the relative importance of substrate type and larval density in bacterial community dynamics is unknown. We investigated four larval densities (0 [control], 50, 100, or 200 larvae per container [520 mL; diameter, 75 mm]) and three feed substrates (chicken feed, chicken manure, and camelina substrate [50% chicken feed, 50% camelina oilseed press cake]) and sampled the bacterial communities of the substrates and larvae at three time points over 15 days. Although feed substrate was the strongest driver of microbiota composition over time, larval density significantly altered the relative abundances of several common bacterial genera, including potential pathogens, in each substrate and in larvae fed chicken feed. Bacterial communities of the larvae and substrate differed to a higher degree in chicken manure and camelina than in chicken feed. This supports the substrate-dependent impact of black soldier fly larvae on bacteria both within the larvae and in the substrate. This study indicates that substrate composition and larval density can alter bacterial community composition and might be used to improve insect microbiological safety.

**IMPORTANCE** Black soldier fly larvae can process organic side streams into nutritious insect biomass, yielding a sustainable ingredient of animal feed. In processing such organic residues, the larvae impact the substrate and its microbiota. However, their role relative to the feed substrate in shaping the bacterial community is unknown. This may be important for the waste management industry to determine whether pathogens can be controlled by manipulating the larval density and the timing of harvest. We investigated how the type of feed substrate and the larval density (number of larvae per container) interacted to influence bacterial community composition in the substrates and larvae over time. Substrate type was the strongest driver of bacterial community composition, and the magnitude of the impact of the larvae depended on the substrate type and larval density. Thus, both substrate composition and larval density may be used to improve the microbiological safety of the larvae as animal feed.

## INTRODUCTION

The saprophagous larvae of the black soldier fly (BSF), Hermetia illucens L. (Diptera: Stratiomyidae), are promising agents in the management of organic waste and its conversion into insect biomass for animal feed (1). The larvae can consume a wide range of organic side streams such as manure, sewage sludge, fish offal, kitchen waste, fruits and vegetables, and brewery spent grains (2–4). The larval foraging period takes 15 to 21 days at 28°C (5, 6), and once fully grown, the larval body contains 39 to 63% protein and 7 to 39% fat depending mainly on the feed substrate (1). The larval protein is a valuable ingredient as feed for poultry, pigs, and fish and can be more sustainable than soymeal or fishmeal (7–11). In most bioconversion systems, the larvae interact with a rich microbial community of bacteria (12), fungi (13), viruses (14), archaea (15), and possibly protists (16). The bacterial community may be important for larval performance but can also contain potential pathogens for humans and livestock animals (17, 18). Bacteria may aid in substrate digestion and provide essential nutrients to the insect host but may also compete for nutrients (19, 20). For example, in previous experimental studies, the BSF larval growth rate increased when the feed substrate was inoculated with Bacillus subtilis or *Arthrobacter* sp. strain AK19 but decreased when inoculated with Bifidobacterium breve (21, 22).

The bacterial community composition of the larval gut is determined mainly by the feed substrate (12, 23). The gut microbiota reflects a shrinking subset of substrate-associated bacteria as digestion progresses through the midgut (23). The larval gut poses a strong selection pressure on ingested bacteria due to the production of a range of lysozymes and antimicrobial peptides and steep pH gradients going from pH 6 to pH 2 to pH 8 in the anterior, middle, and posterior midguts, respectively (24, 25). The majority of ingested bacteria are thus digested, and only a subset survives and may reproduce in the gut (23). Despite diet-dependent differences in the bacterial community composition of larvae, some bacterial taxa can be shared across larvae fed different substrates (12, 15, 18) and may comprise a core microbiota of BSF (26).

Similar to some other saprophagous fly species, BSF larvae impact substrate bacterial communities and physicochemical properties through digestion, immune defense, and larval aggregation (27). The foraging of BSF larvae in an aggregation, or maggot mass, changes the substrate pH to 8 to 9 regardless of the initial pH (28), decreases the manure moisture content and the emission of microbial volatiles such as indole (29), and reduces populations of Escherichia coli and Salmonella spp. (30). This impact of larvae on the substrate likely increases with larval age and size and is therefore time dependent (18).

**FIG 1:**
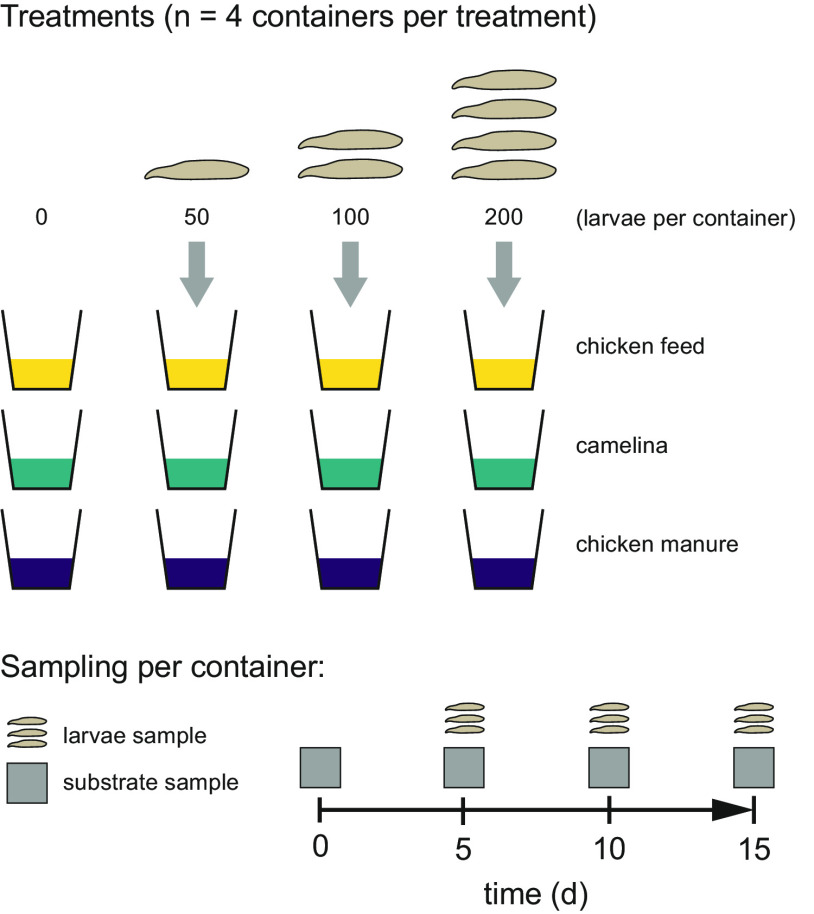
Experimental treatments of the study. We tested the effect of larval density (0, 50, 100, and 200 larvae per container) on the substrate and larval microbiota in three feed substrates (chicken feed, camelina, and chicken manure) (*n* = 4, i.e., 4 biological replicates [containers] per treatment group [substrate × density combination]). Per container, the substrate microbiota was sampled on days 0 (i.e., the start of the experiment), 5, 10, and 15, and larval microbiota were sampled on days 5, 10, and 15 (in treatments with larvae). Larval performance parameters and substrate samples for pH and moisture content were collected upon the termination of the experiment on day 15.

Although BSF larvae can impact the substrate and its microbiota, their role relative to the feed substrate in shaping the bacterial community composition remains to be investigated. The effect of BSF larvae on their internal and substrate microbiota may be important in the waste management industry, for instance, to determine whether potential pathogens can be controlled by manipulating the larval density and the timing of harvest (17, 26). In this study, we aimed to elucidate the relative importance of larval density and substrate type in structuring the bacterial community composition in the substrate and larval gut. We tested four different larval densities (0 [control], 50, 100, or 200 larvae per container [520 mL; diameter, 75 mm]) on three feed substrates (chicken feed, chicken manure, and camelina substrate [50% chicken feed, 50% camelina oilseed press cake]) and sampled the bacterial communities of the substrates and larvae over time (Fig. 1).

## RESULTS

### Larval performance.

Larvae performed differently on the three substrates, and individual larval weights decreased with higher larval densities in two substrates (Table 1). Survival rates differed among substrates but not among larval densities (main effect of substrate, *P < *0.001; main effect of density, *P *= 0.296 [by generalized least-squares {GLS} regression]). More larvae survived on the camelina substrate (88 to 92%) than on the other two substrates (chicken feed, 60 to 66%; chicken manure, 69 to 84%). At day 15, in chicken manure and the camelina substrate, significantly fewer larvae had developed into the prepupal stage (9 to 16% and 0 to 2%, respectively) than in chicken feed (86 to 98%) (main effect of substrate, *P < *0.001 [by GLS regression]). The larval density negatively affected individual larval weights in chicken manure and chicken feed (main effect of substrate, *P < *0.001; main effect of density, *P < *0.001 [by generalized linear model {GLM} regression]). In chicken manure, individual larval weights differed among all larval densities (50 larvae per container, 70 mg dry matter, 100 larvae per container, 44 mg; 200 larvae per container, 25 mg), and in chicken feed, the larvae at the highest density (200 per container, 55 mg) were smaller than those at the lowest density (50 per container, 81 mg).

**TABLE 1:** Larval performance parameters and substrate properties on day 15^*a*^

Feed substrate	Larval density (no. of larvae)	Mean survival rate (%) ± SE	Mean % prepupae ± SE	Mean individual larval wt (mg dry matter) ± SE	Mean substrate moisture content (% fresh matter) ± SE	Mean substrate pH ± SE
Chicken feed	0				56.7 ± 2.1 A	7.20 ± 0.30 CD
	50	65.9 ± 7.5 ABC	86.0 ± 5.0 B	81.3 ± 5.6 D	64.4 ± 2.1 AB	7.49 ± 0.30 CD
	100	66.5 ± 7.5 ABC	90.6 ± 5.0 B	72.8 ± 5.0 CD	62.3 ± 2.1 A	8.05 ± 0.30 DEF
	200	60.4 ± 7.5 AB	97.7 ± 5.0 B	54.9 ± 3.8 BC	61.0 ± 2.1 A	8.22 ± 0.30 DEF

Camelina	0				73.1 ± 0.4 C	6.55 ± 0.28 BC
	50	88.4 ± 1.6 BC	2.0 ± 1.4 A	66.6 ± 4.6 CD	75.1 ± 0.4 CD	5.17 ± 0.28 A
	100	91.8 ± 1.6 C	1.3 ± 1.4 A	72.2 ± 5.0 CD	76.9 ± 0.4 D	5.55 ± 0.28 AB
	200	92.4 ± 1.6 C	0.0 ± 1.4 A	61.7 ± 4.3 CD	80.2 ± 0.4 E	8.44 ± 0.28 DEF

Chicken manure	0				71.7 ± 1.0 BC	8.79 ± 0.09 EF
	50	82.3 ± 4.7 ABC	8.5 ± 5.3 A	70.4 ± 4.8 CD	72.7 ± 1.0 BC	8.67 ± 0.09 E
	100	69.2 ± 4.7 A	9.1 ± 5.3 A	44.0 ± 3.0 B	71.4 ± 1.0 BC	8.89 ± 0.09 EF
	200	84.2 ± 4.7 ABC	15.8 ± 5.3 A	24.5 ± 1.7 A	73.1 ± 1.0 BCD	9.07 ± 0.09 F

a Shown are estimated marginal means ± standard errors (SE) (*n* = 4). Within a column, means with no shared letters are significantly different (α = 0.05 by Tukey contrasts). Statistical testing was done using GLS (survival, prepupae, and moisture), gamma GLM (individual larval weight), and LMM (pH) regressions.

### Substrate pH and moisture content.

The pH of the fresh feed substrates differed (camelina, pH 5.4; chicken feed, pH 6.3; chicken manure, pH 7.7 [*P* = 0.002 by linear mixed-model {LMM} regression]). The substrate pH on day 15 of the experiment also showed differences among the substrates and an effect of larval density on the camelina substrate and chicken manure (main effect of substrate, *P < *0.001; main effect of density, *P < *0.001; interaction of substrate × density, *P < *0.001 [by LMM]) (Table 1). The pH of chicken manure was 8.7 to 9.1, and the substrate pH at a larval density of 50 larvae per container was lower (pH 8.7) than that at 200 larvae per container (pH 9.1). The chicken feed pH ranged from 7.2 to 8.5, and the camelina substrate pH ranged from 5.2 to 8.4. In camelina, the pH was the lowest at larval densities of 50 and 100 larvae per container (pH 5.2 and 5.5, respectively), intermediate in substrates without larvae (pH 6.6), and highest in substrates with 200 larvae per container (pH 8.4). The substrate moisture content on day 15 did not differ among larval densities in chicken feed (57 to 64%) or chicken manure (71 to 73%), but in camelina, it increased with larval density (from 73% to 80%) (Table 1).

### 16S rRNA gene amplicon sequencing quality control.

The bacterial community composition was assessed by PCR amplification and sequencing of the V5-V6 variable region of the 16S rRNA gene. This resulted in 68 million reads (after the removal of mitochondrial and chloroplast DNAs). The day 0 substrate samples of camelina were 100% mitochondrial and chloroplast DNAs and therefore were excluded after this step. No-template controls (NTCs) for PCR contained 3,433 to 11,485 reads, belonging to 28 genera (see Table S1 in the supplemental material), whereas the sequencing depth of samples ranged from 8,154 to 493,735 reads. Seven samples contained fewer reads than the highest sequencing depth of a no-template control; they all belonged to the chicken feed substrate samples at day 0. Twenty-six amplicon sequence variants (ASVs) were identified as contaminants and removed from the data set (Table S2), mainly concerning known laboratory contaminants (31). Further analyses were performed on relative abundance data at the genus level, where relative abundance is the number of reads of a genus in a sample divided by the total number of reads in that sample. Read abundances in positive controls, i.e., synthetic mock communities of known composition (two different controls, mock 3 and mock 4) (32), showed a high correlation with the theoretical mock community composition (mock 3, Spearman correlation [*r*] = 0.78 to 0.87; mock 4, *r* = 0.68 to 0.77) and between technical replicates of different sequencing libraries (mock 3, *r* = 0.92 to 0.99; mock 4, *r* = 0.95 to 0.99). Read abundances in technical replicates of DNA isolation of the substrate microbiota were highly correlated (Spearman *r* = 0.73 to 0.94), with a few exceptions (*r* = 0.38, 0.47, and 0.60 for chicken feed with 200 larvae per container on day 5, chicken feed without larvae on day 15, and the camelina substrate without larvae on day 5, respectively), as were technical replicates of PCR across sequencing libraries (Spearman *r* = 0.88 to 0.98).

### Alpha diversity of substrate and larval microbiota.

**FIG 2:**
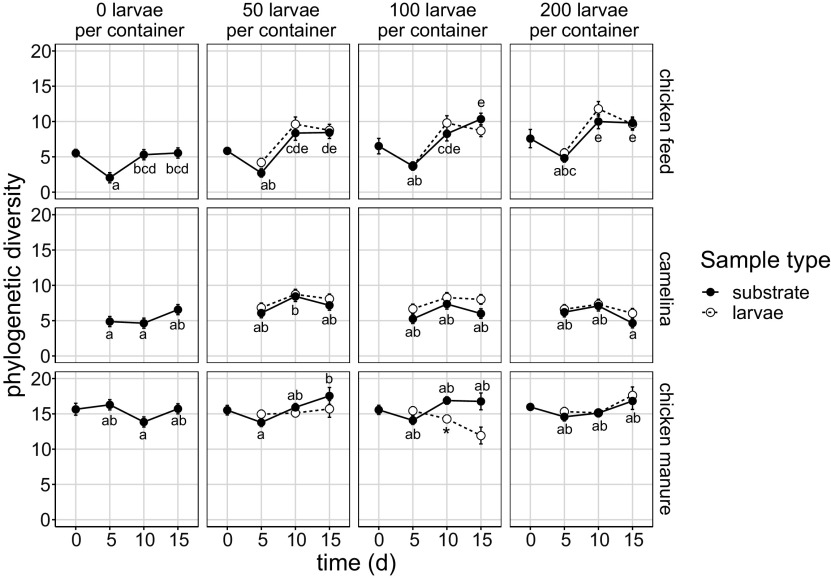
Faith’s phylogenetic diversity (estimated marginal means ± SE) (*n* = 4) of the larval and substrate microbiota over time, in chicken feed (top row), camelina (middle), and chicken manure (bottom), separated by larval density. Within a diet (row of four panels), means of substrate microbiota diversity with no shared letters are significantly different (α = 0.05 by Tukey contrasts from LMM regression on substrate samples, excluding day 0); means of larval microbiota diversity with an asterisk are significantly different from those of the corresponding substrate microbiota (α = 0.05 by Tukey contrasts from LMM regression on substrate and larval samples, excluding samples of day 0 or 0 larvae per container).

Alpha diversity was measured using Faith’s phylogenetic diversity. The substrate microbiota (excluding day 0) of chicken manure was more diverse than that of the other substrates (main effect of substrate, *P < *0.001 [by LMM]) (Fig. 2). Over time, diversity did not change in the camelina or chicken manure substrates, except for an increase in diversity in chicken manure with 50 larvae per container. However, in chicken feed substrates, diversity increased from days 5 to 15 (main effect of time, *P < *0.001; interaction of substrate × time, *P < *0.001) (*post hoc* test results are reported in Fig. 2). Moreover, in chicken feed substrates with 100 or 200 larvae per container, diversity was higher than that in the substrate without larvae (main effect of density, *P < *0.001; interaction of substrate × density, *P* = 0.004; interaction of density × time, *P* = 0.001). The larval and substrate bacterial diversity differed only in chicken manure with 100 larvae per container on day 10 (Fig. 2).

Patterns of Shannon diversity were comparable to those of Faith’s phylogenetic diversity, except for the diversity of the substrate microbiota in camelina, which decreased over time in the presence of larvae, and for larval microbiota that were more diverse than the substrate microbiota on day 15 in the camelina substrate with 100 larvae per container (Fig. S1).

### Effects of feed substrate and larval density on substrate microbiota.

**FIG 3:**
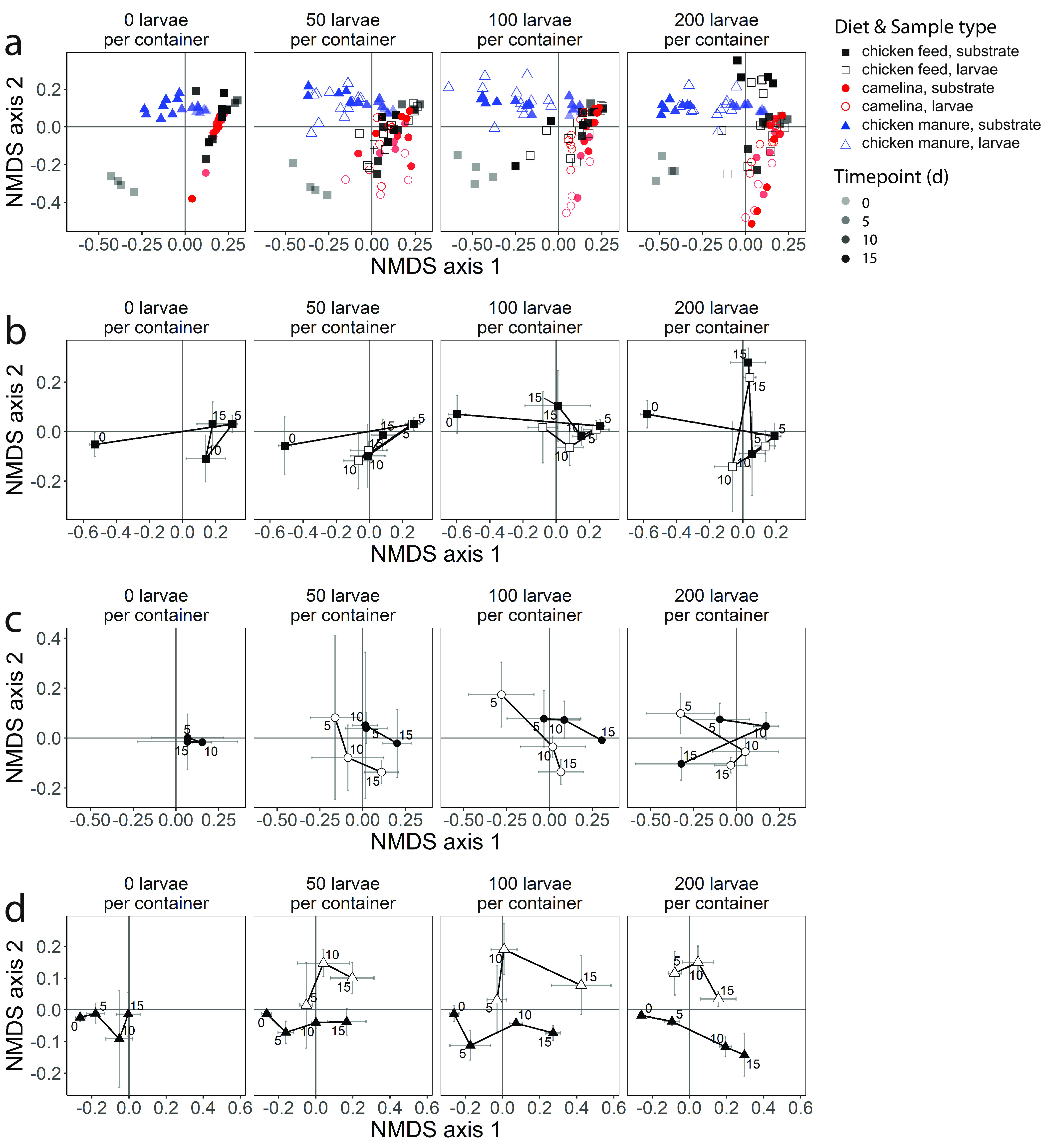
Microbiota compositions of larvae and substrates (NMDS of weighted UniFrac distances). (a) All three feed substrates combined; (b) chicken feed; (c) camelina; (d) chicken manure. Plots show microbiota variation along the 1st and 2nd NMDS axes. Panels a to d are separate NMDS ordinations; i.e., in panels b to d, the NMDS is done only on samples of the respective feed substrates. Each row is one ordination split into four panels for visibility, corresponding to the four larval densities (0, 50, 100, or 200 larvae per container). Colors in panel a represent chicken feed (black), camelina (red), and chicken manure (blue). In panel a, individual replicates (containers) are plotted, with time points (days) displayed as a transparency gradient (with day 0 being the most transparent and day 15 being nontransparent). In panels b to d, time points are labeled in the plot. Error bars in panels b to d are means ± standard deviations (SD) of axis scores (*n* = 4). Values for stress of NMDS solutions are 0.129 (a), 0.090 (b), 0.125 (c), and 0.133 (d).

**FIG 4:**
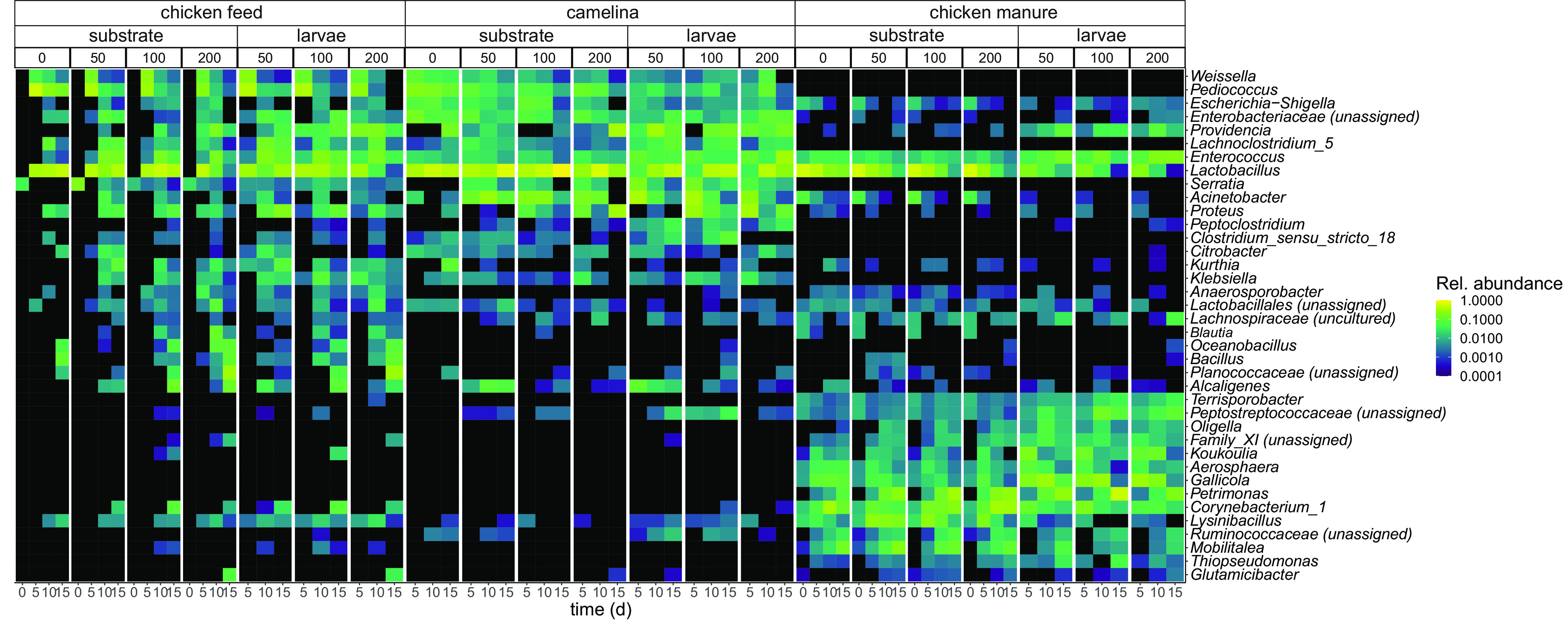
Heat map of the most abundant bacterial genera over time. Only genera with an overall maximum relative abundance of >0.1 and present in >10% of the samples are displayed. Mean relative abundance (*n* = 4) is displayed by the color scale; black indicates a mean relative abundance of zero. The plot is divided into panels for the feed substrate (chicken feed, camelina, or chicken manure), sample type (substrate or larvae), and larval density (0, 50, 100, or 200 larvae per container) (top of the plot), with data separated per time point (in days) along the *x* axis within each panel.

The bacterial community composition of substrates differed among substrates and over time (nonmetric multidimensional scaling [NMDS] of weighted UniFrac distances) (Fig. 3a), with *Firmicutes*, *Proteobacteria*, *Actinobacteria*, and *Bacteroidetes* being the most predominant phyla (Fig. S2). The substrate × density × time model explained 75% of the total microbiota variation (distance-based redundancy analysis [dbRDA]) (Table S3). The main effect of substrate explained the majority of microbiota variation (38%), followed by the effects of time within each substrate (substrate × time, 11%) (permutational multivariate analysis of variance [PERMANOVA]) (Table S3). The microbiota of all three substrates differed from each other. The chicken manure substrate microbiota was most distinct from those of the other two substrates (*P* = 0.001 [by PERMANOVA pairwise contrast]) (Fig. 3a). Substrates of chicken manure contained 12 abundant genera that were virtually absent in the other substrates, including *Petrimonas*, *Gallicola*, *Koukoulia*, *Aerosphaera*, and an unassigned genus of *Clostridiales* family XI (Fig. 4). Chicken manure also virtually lacked eight genera that were abundant in the other substrates, including Klebsiella, *Weissella*, *Serratia*, *Pediococcus*, and *Lachnoclostridium*_*5* (Fig. 4).

Time explained most variation in the substrate microbiota in each substrate, followed by larval density (dbRDA and PERMANOVA) (Table S4). Within each substrate, there were differences among larval densities in the substrate bacterial community composition (Fig. 3b to d). The chicken feed microbiota were initially (day 0) rich in *Curtobacterium* and *Pantoea* (not included in Fig. 4 because of selection thresholds) but changed drastically to a dominance of the lactic acid-producing bacteria *Pediococcus*, *Lactobacillus*, and *Weissella* 5 days later (Fig. 4). In chicken feed, the substrate microbiota with 200 larvae per container differed significantly from those without or with 50 larvae per container on day 15 (Fig. 3b; Fig. S3a) (distance-based principal-response curves [dbPRCs] with *post hoc* Tukey contrasts). Relative to the control without larvae on day 15, the camelina substrate microbiota with 50 or 100 larvae per container differed from the substrate microbiota with 200 larvae per container (Fig. 3c; Fig. S3b). In chicken manure, the substrate microbiota with 100 or 200 larvae per container started to differ on day 15 or 10, respectively, from the substrate microbiota without or with 50 larvae (Fig. 3d; Fig. S3c). This indicated that the change in the chicken manure substrate microbiota happened at a higher rate with higher larval densities.

Substrates with lower larval densities had higher relative abundances of lactic acid-producing bacteria (*Lactobacillus* and *Pediococcus*) (dbPRC) (Fig. S3). Apart from these shared patterns, different genera were increased in relative abundance with larval density in each feed substrate (dbPRC) (Fig. S3): *Providencia* and Proteus in the camelina substrate, *Petrimonas* and *Corynebacterium_1* in chicken manure, and an unassigned genus of *Planococcaceae* in chicken feed.

### Effects of feed substrate and larval density on larval microbiota.

Similar to what was found for the substrate microbiota, the type of feed substrate and time were the main drivers of the larval microbiota (Table S5). Larval density significantly influenced the internal microbiota in larvae fed chicken feed but not in larvae fed camelina or chicken manure (Table S6 and Fig. S4 [*post hoc* test results are included in Fig. S4]). In chicken feed, the larval microbiota significantly differed between the density of 200 larvae per container and the lower larval densities: at 200 larvae per container, the larvae had higher relative abundances of, e.g., *Planococcaceae* (unassigned genus), *Bacillaceae* (uncultured), and *Bacillus* and lower relative abundances of, e.g., *Lactobacillus*, Proteus, and *Enterococcus* (dbPRC) (Fig. S4).

The similarity between the larval and substrate microbiota depended on the feed substrate (Fig. S5). The larval and substrate microbiota in chicken feed did not differ significantly (Fig. S5). Nonetheless, across larval densities, larvae tended to be more associated with *Providencia*, whereas the substrates tended to be more associated with *Lactobacillus* (dbPRC) (Fig. 4; Fig. S5).

In camelina, the microbiota of the larvae and substrates developed differently over time (Fig. 3c). The larval and substrate microbiota differed in composition at a larval density of 100 larvae per container on day 15, but they overlapped at the other densities (Fig. 3c). At 100 larvae per container, larvae were associated with *Providencia* and Proteus, whereas the substrates were more associated with *Lactobacillus* (dbPRC) (Fig. 4; Fig. S5).

In chicken manure, the larval and substrate microbiota were clearly separated at all densities (Fig. 3d). Across larval densities, larvae were more associated with *Providencia*, an unassigned genus of *Peptostreptococcaceae*, *Gallicola*, *Enterococcus*, and *Koukoulia*, whereas the substrates were more associated with *Lactobacillus*, *Mobilitalea*, *Lysinibacillus*, *Corynebacterium_1*, and *Petrimonas* (dbPRC) (Fig. 4; Fig. S5).

### Microbial growth on the substrate surface.

On the surface of the feed substrates, extensive growth of microbes was observed, the appearance of which differed between chicken feed on the one hand and chicken manure and camelina on the other hand. These surface layers were not investigated in a systematic way, and we did not investigate if these layers meet the definition of biofilms (33). We photographed all containers on day 6 for batch 1 and day 5 for batch 2 and a few containers also on later days. In chicken feed, all containers had fungal overgrowth with sporulating structures on top of the substrate on day 5/6. In only some containers (not systematically recorded) did larvae manage to disintegrate this fungal layer over time. In camelina, on day 5/6, the substrates had a layer with a slimy appearance, likely consisting of bacteria and fungi. The bacterial community composition of this layer was variable and dominated by one or more of the following bacterial taxa: Acinetobacter, *Serratia*, *Comamonas*, and/or *Enterobacteriaceae* (Fig. S6). *Lactobacillus* was less abundant in these biofilms than in the substrate beneath. Larvae disintegrated these biofilms over time, and they had disappeared on day 15 at the high larval densities (100 to 200 larvae). In chicken manure, the substrate surface also carried a top layer of slime-producing bacteria on day 5/6. By day 10, larvae had disintegrated this layer.

## DISCUSSION

Black soldier fly larvae are voracious consumers of decomposing organic matter, and in their foraging, they interact with dense bacterial populations in the feed substrate that colonize the larval gut. This study presents the magnitude of the effects of the larvae and feed substrate on the development of the internal and substrate microbiota over time. The bacterial community composition in the substrates was driven mainly by the type of feed substrate and the density of BSF larvae. In addition, the larvae and substrates differed in their microbiota compositions, depending on the feed substrate and larval density.

### Feed substrate is the main driver of microbiota variation.

The largest driver of variation in the microbiota of both the larvae and substrates was the type of feed substrate. The chicken manure microbiota differed considerably from the microbiota of chicken feed and the camelina substrate. These differences are likely related to nutrient composition (5, 34, 35) and substrate origin (26). The camelina substrate shared many bacterial genera with chicken feed, but these genera differed in relative abundances between the two substrates. The overlap in genera is most likely the result of the camelina substrate containing 50% chicken feed. The differences in relative abundances may be caused by nutrient composition and moisture content (34), crop-associated bacteria (36), and isothiocyanates from the camelina press cake (37). Isothiocyanates are derivatives of secondary plant compounds (glucosinolates) of crucifer crops, which have antimicrobial effects (38).

### Substrate-dependent impact of larval density on substrate microbiota.

The larval density significantly altered the bacterial community composition in all three feed substrates. With increasing larval density, *Lactobacillus* decreased across substrates, whereas different genera increased depending on the type of feed substrate. These changes might be caused by larval foraging in maggot masses. Maggot mass foraging generally increases the local peak temperature, aeration, and pH of the substrate and decreases its moisture content (28, 29, 39–41). In addition, the maggot mass increases the decomposition rate (40) and alters microbial metabolism and the resultant volatile emissions (29). We also found that BSF larvae affect bacterial alpha diversity differently depending on the feed substrate, whereas larvae of the housefly Musca domestica L. and the fruit fly Drosophila melanogaster (Meigen, 1830) decrease bacterial and yeast diversity, respectively (27, 42, 43). Housefly larvae possibly decrease bacterial diversity in swine manure by the rapid consumption of the substrate (42), and fruit fly larvae may decrease yeast diversity by encouraging the growth of only a few palatable yeast species (43).

BSF larval density influenced the relative abundances of potentially pathogenic bacteria over time. In camelina substrates and larvae, *Providencia* and Proteus increased in relative abundance with larval density; *Serratia* and Acinetobacter, however, decreased over time. *Corynebacterium_1* increased with larval density in chicken manure substrates. Note, however, that (i) based only on 16S rRNA amplicons, we could not confirm if the identified sequences belonged to pathogenic strains and (ii) the observed patterns consider compositional data and can give insights into bacterial population sizes only if supplemented with data on absolute abundance through quantitative PCR.

BSF larvae alter the bacterial community composition of substrates by introducing gut-associated bacteria (40) and/or changing the population sizes of resident bacteria in the substrate (30, 44, 45). Gut-associated bacteria can make up 66% of the substrate microbiota after 2 days of larval feeding (starting with 5-day-old larvae), before gradually decreasing to 13% on the 10th day (40). In previous studies, larvae 10 to 15 days old decreased Salmonella and E. coli populations (log_10_ CFU per gram) in contaminated manure after three or more days of feeding, compared to a control without larvae (30, 44, 45). In our study, larvae significantly altered the substrate microbiota on days 10 to 15. Administering the substrate at intervals, i.e., adding fresh substrate every few days, instead of in bulk from the start as applied here might offset such an impact of larvae on the substrate microbiota (e.g., see reference 23).

The impact of larvae on the bacterial community composition was different in each substrate, possibly related to larval performance, foraging behavior, and substrate nutrient composition and concentration. The lower larval weight at a higher larval density in chicken feed and chicken manure may indicate a food shortage, which can drastically impact the BSF larval gut microbiota (46) and might have effects on larval foraging behavior and the substrate microbiota as well. Substrate nutritional quality, moisture content, and initial pH influence larval performance (28, 39, 47, 48). Additionally, the initial microbiota of the substrate may affect larval performance (26), and the substrate may alter the larval immune response and larval digestion (24, 49). This might cause the substrate-dependent impact of larvae on substrate microbiota composition. The low larval survival in chicken feed may have resulted from fungal overgrowth on this substrate, which could cause high mortality among first-instar larvae (50).

The resident microbiota of the three substrates may have been differentially altered due to differences in larval development rates and associated foraging behaviors. Once prepupae, larvae cease feeding (51, 52) and likely have a smaller impact on the substrate and its microbiota than the penultimate larval instar. Larvae fed chicken manure or camelina developed significantly more slowly than larvae fed chicken feed. Such differences in larval development might be related to the initial nutrient composition of the feed substrate (2, 5, 6), which changes over time due to the accumulation of frass.

The high pH of all substrates on day 15 likely resulted from proteolysis and the accumulation of ammonia (53). The lower pH of substrates with 50 or 100 larvae per container in the camelina substrate might be related to the increase in *Lactobacillus* in this substrate over time, as opposed to the other substrates. Furthermore, most larvae in the camelina substrate initially foraged in the surface layer, in which we demonstrated high relative abundances of Acinetobacter, *Serratia*, and *Comamonas*, and moved deeper into the substrate only after 5 to 7 days. This behavior was observed in deeper substrate layers at higher larval densities, which might have increased substrate aeration and enhanced aerobic microbial metabolism (41). Consequently, lactic acid fermentation could have diminished, and this resulted in a higher substrate pH only at the highest larval density. The surface layers on top of the substrates were avoided for substrate sampling because they likely contained a higher population density of bacteria and/or a different community composition than that of the underlying substrate. The possible involvement of biofilms and their effects could have influenced the results in several ways, as discussed in Text S1 in the supplemental material.

### Larval microbiota composition was determined by feed substrate and larval density.

The composition of the larval microbiota was determined primarily by the feed substrate, as was found previously (12, 15, 23, 54), and, to a lesser extent, by larval density. The larval density significantly influenced the larval microbiota composition in larvae fed chicken feed, with decreases in the relative abundances of the potentially pathogenic bacteria *Enterococcus*, Proteus, *Providencia*, and *Enterobacteriaceae* (unassigned) and an increase in *Bacillus* (55). Although the larval and substrate microbiota mostly developed in a similar way over time, their compositions differed significantly in chicken manure and the camelina substrate (the latter at 100 larvae per container, on day 15). This may be because the type of substrate influences the larval immune response and digestive function (24, 25, 54, 56), resulting in substrate-dependent selection pressure of larvae on ingested and resident gut bacteria (23). Bonelli et al. (56) showed that in response to different feed substrates, BSF larvae have different activities of digestive enzymes, morphologies of the midgut epithelia, and gene expression related to digestion or absorption. In addition, Vogel et al. (24) found that the larval immune response is stronger in BSF larvae fed substrates with a high bacterial load or supplemented with protein or plant oils. Hence, chicken manure and the camelina substrate may have triggered a more complex and stronger larval immune response than chicken feed since chicken manure has a high bacterial load and the camelina substrate is rich in protein (32% dry matter) and camelina seed oil (8% dry matter) (5). Bacteria that survive the conditions of the larval gut may proliferate and become more abundant in the larva than in the surrounding substrate. This might apply to *Providencia* in larvae fed any of the three substrates, Proteus in larvae fed camelina, and *Gallicola* and *Enterococcus* in larvae fed chicken manure. However, a higher relative abundance could also represent a lower total absolute abundance of bacteria.

*Providencia*, *Lactobacillus*, and *Enterococcus* persisted in larvae across feed substrates and time. Some studies identify the shared bacterial taxa across substrates as a core microbiota of BSF larvae that may confer benefits to host functioning and survival (12, 15, 18, 54). *Providencia* may be transmitted vertically from adult females to eggs (57), and a strain of this genus has been isolated from eggs of our BSF colony, along with a strain of *Lysinibacillus* (S. J. J. Schreven, unpublished data). Several egg-associated bacteria, e.g., Enterococcus faecalis and Lysinibacillus boronitolerans, increase the BSF egg-hatching rate, the larval growth rate, and/or adult female fecundity (22, 58–62). Although *Providencia* and *Enterococcus* have been identified as core taxa of BSF larvae in other studies, there is considerable variation in the identified core among studies (12, 18). For instance, *Dysgonomonas*, *Parabacteroides*, Pseudomonas, and *Morganella* have been reported as core taxa of BSF larvae, but in our study, *Morganella* was absent, and the other three were rarely present (12, 18, 40, 63). This variability may be due to the rearing facility and host genotype but may also be because the identified core in some studies could include the microbiota of a nursery substrate (18, 64). In our study, we found different taxa enriched in larvae depending on the substrate. The core microbiome of BSF larvae may be defined by critical functions (i.e., the functional core microbiome [65]) rather than specific taxa, and BSF larvae may be able to select for those (24). Identification of these critical gut microbiome functions that complement host function may result in an understanding of BSF microbial ecology that is more applicable to the edible-insect industry.

### Conclusion.

BSF larvae altered the bacterial community composition in all three substrates. This effect was different in each substrate and also dependent on larval densities. Although the impact of larval density was subordinate to the effects of the feed substrate, larval density significantly altered the relative abundances of some of the most common bacterial genera, including potential pathogens, in each substrate and in larvae fed chicken feed. Remarkably, the larval and substrate microbiota were distinct in chicken manure and the camelina diet, whereas they overlapped in chicken feed. These findings highlight the flexible associations between bacteria and BSF larvae and support the concept of substrate-dependent selection of bacteria by BSF larvae. For the edible-insect industry, our study indicates that substrate composition and larval density can alter microbial community composition and possibly improve insect microbial safety.

## MATERIALS AND METHODS

### Insects.

Insects originated from the black soldier fly colony of the Laboratory of Entomology, Wageningen University, The Netherlands. The colony was established with source material from the United States around 2008 and has since been reared at 27°C ± 2°C with 70% ± 10% relative humidity and a photoperiod of 14 h of light/10 h of darkness (L14:D10). The larvae were reared on chicken feed (Kuikenopfokmeel 1; Kasper Faunafoods BV, The Netherlands). Eggs <24 h old were collected in cardboard strips deployed in the adult cage and incubated under the same abiotic conditions, and neonate larvae were transferred to treatment feed substrates within 24 h after hatching.

### Feed substrates.

Three experimental substrates were used. Chicken feed was the same as the colony substrate; organic chicken manure free of antibiotics and pesticides was collected freshly from a belt system of layer hens at Carus experimental farm (Wageningen University) and used in the experiment on the same day. The camelina substrate was a 1:1 mixture on a dry matter basis of chicken feed-Camelina sativa press cake. The press cake was produced from mechanical pressing of seeds (produced without the application of insecticides) from the 2015 harvest of the University of Warmia and Mazury in Olsztyn, Poland. Substrates were prepared with 36.0 g dry matter of feed and 67.1% moisture (chicken feed) or 78.6% moisture (camelina substrate, controlled for the higher water retention capacity than that of chicken feed) or with 46.6 g dry matter of feed and 75.4% moisture (fresh chicken manure, with no water added).

### Experimental design.

To determine the influence of larval density (0, 50, 100, or 200 neonate larvae per container) on bacterial community dynamics in three different substrates (chicken feed, 50% camelina press cake, and chicken manure) over the course of 15 days, we used four replicates per treatment, divided into two batches of two replicates each, started on consecutive days. The experiment was conducted in a climate chamber at 27°C ± 2°C with 70% ± 10% relative humidity and an L14:D10 photoperiod, from 15 June to 1 July 2017. Every day, the position of each container was randomly changed to control for any abiotic gradient in the climate chamber. Containers were Superfos UniPak 5012 polypropylene transparent containers with a 520-mL volume, a bottom diameter of 75 mm, and a top diameter of 95 mm (RPC Superfos, Taastrup, Denmark), with a mesh lid (mesh area of 60 mm in diameter; ~0.5-mm mesh size). Containers were disinfected with 70% ethanol prior to use.

### Performance parameters and substrate pH.

On day 15, the experiment was terminated, and larvae were harvested. Larvae were counted to determine the survival rate, rinsed with lukewarm tap water, gently dried using paper tissues, weighed as fresh biomass yield (Ohaus Adventurer Pro AV313 [precision = 0.001 g]; Ohaus Corp. USA), and then frozen at −21°C. The proportion of prepupae was determined using the degree of dark pigmentation of the cuticle. A subsample of 10 larvae from each container was dried in a stove at 70°C until a stable weight was reached (Mettler-Toledo ML54/01), to determine the dry matter content and dry larval biomass. Substrates were stored at −21°C, and subsamples of 2 to 7 g fresh matter (FM) were oven dried at 70°C until a stable weight was reached (Mettler-Toledo ML54/01). The larval survival rate was calculated as the number of living larvae at the time of harvest divided by the number of larvae on day 0, minus 9 (i.e., the number of larvae collected for analysis, 3 larvae at three time points).

The substrate pH of samples on day 15 was measured in a suspension of approximately 1 g FM of the harvested substrate (weighed at 0.0001 g precision) (Mettler Toledo ML54/01) in 10 mL MilliQ water (Merck KGaA, Darmstadt, Germany). Within an hour, the pH was measured using a pH meter (ProLine B210; ProSense BV, The Netherlands). After the experiment, the pH of the newly prepared substrates of chicken feed and camelina press cake and of the fresh chicken manure batches (four batches, one per replicate), of which reference material had been stored at −21°C, was measured in triplicate using the same method.

### DNA sample collection.

For DNA isolation, substrate samples were collected on days 0, 5, 10, and 15; larval samples were collected on days 5, 10, and 15. On day 0, substrate samples were collected 1 h after the distribution of the substrate into the containers and the addition of water to the substrate and prior to the addition of larvae. Substrate samples were taken by removing the top layer (top 1 to 5 mm) of the substrate and then taking a sample of the full depth of the substrate using a sterile plastic straw (7-mm diameter). This sample was then placed into a 1.5-mL tube and mixed thoroughly for 30 s using a small spatula. For each larval sample, three larvae were surface sterilized in petri dishes using the same rinsing protocol as the one described previously by Schreven et al. (66): MilliQ water (30 s), 70% ethanol (30 s), 1% Halamid-D (chloramine-T) (20 s), and two 10-s rinses in MilliQ water. The three larvae were then placed into a 1.5-mL tube. The substrate and larval samples were snap-frozen in liquid nitrogen and then stored at −80°C.

In addition, the top layer of some substrates on day 5 was sampled using a sterile spatula and scraping the top 1 to 5 mm of the substrate for composition analysis of biofilms; the downstream processing of these biofilm samples was the same as that for the substrate samples.

### Sample homogenization and DNA isolation.

The methods for cell lysis, repeated bead beating, and subsequent DNA extraction were adapted from the ones described previously by Salonen et al. (67) and Van Lingen et al. (68), according to the same procedure as the one used by Schreven et al. (66). Larval samples were homogenized in 300 μL buffer for stool transport and recovery (STAR; Roche) in sterile 2.0-mL screw-cap tubes with 0.25 g of 0.1-mm zirconia beads and 3 glass beads (2.5 mm). Small larvae were homogenized per pool of three larvae using a bead beater (Precellys 24; Bertin Technologies, France) at room temperature at 5.5 m s^−1^ three times for 1 min with 20-s intervals, incubated in a shaker for 15 min at 95°C at 300 rpm, and centrifuged for 5 min at 4°C at 16,100 × *g*. Large larvae harvested on day 15 were homogenized individually, and prior to homogenization, these frozen larvae were cut with a disinfected spatula behind the mesothoracal segment and before the second-last abdominal segment to facilitate tissue destruction. The supernatant was transferred to a new 2.0-mL tube, and bead beating, incubation, and centrifugation were repeated with 200 μL of STAR buffer to yield a total of approximately 500 μL of the supernatant. From this, 250 μL was transferred to a cartridge of a customized DNA isolation kit (Maxwell 16 tissue LEV total RNA purification kit, catalog no. XAS1220; Promega Corporation, USA), and DNA was isolated and eluted in 30 μL nuclease-free water using the Maxwell MDx robot (Promega Corporation, USA). The three supernatants of larvae from a single sample (container) were pooled in the cartridge using 83 μL of each supernatant (total of 250 μL).

Substrate samples were homogenized in 700 μL STAR buffer (Roche) in sterile 2.0-mL screw-cap tubes with 0.5 g of 0.1-mm zirconia beads and five glass beads (2.5 mm). The samples (0.25 g) were then homogenized in a bead beater at room temperature three times for 1 min at 5.5 m s^−1^ with a waiting step of 20 s in between, incubated in a shaker for 15 min at 95°C at 300 rpm, and centrifuged for 5 min at 4°C at 16,100 × *g*. The supernatant was transferred to a new 2.0-mL tube, and the steps were repeated with 300 μL of STAR buffer to yield a total of approximately 1 mL of the supernatant. The DNA isolation procedure was the same as the one described above for larval samples (250 μL of the supernatant per cartridge).

### Microbiota profiling.

The procedure described below is largely the same as the one described previously by Schreven et al. (66). DNA concentrations were measured with a NanoDrop ND-1000 spectrophotometer (NanoDrop Technologies Inc., Wilmington, DE, USA), and samples were diluted to 20 ng DNA μL^−1^ prior to PCR. The V5-V6 region of the 16S rRNA gene was amplified using barcoded primer pair F784-1064R (32) according to the following PCR program: 98°C for 30 s and 25 cycles of 98°C for 10 s, 42°C for 10 s, 72°C for 10 s, and 72°C for 7 min. Per reaction, the following 50-μL mix was prepared: 36.5 μL of nuclease-free water, 10 μL of 5× Phusion HF buffer (Thermo Fisher Scientific, USA), 1 μL of deoxynucleotide triphosphates (dNTPs) (10 mM), 0.5 μL of Phusion Hot Start II DNA polymerase (2 U μL^−1^) (Thermo Fisher Scientific, USA), 1 μL of barcoded primers (10 μM), and 1 μL of the DNA template. Samples were amplified in duplicate. As positive controls, we used synthetic mock communities of known composition and comprising full-length 16S rRNA gene amplicons of bacterial phylotypes associated with the human gut (32). No-template controls (NTCs) (1 μL nuclease-free water as the template) were included in the PCR. The products were checked for yield and correct size by agarose gel electrophoresis, and PCR amplification was repeated for samples with no or a low yield with 5 μL of the DNA template. PCR products were purified using the CleanPCR magnetic bead suspension (CleanNA, The Netherlands), a 1.8× volume of the PCR mix (duplicates combined), and two washes with 200 μL 70% ethanol and eluted in 30 μL nuclease-free water. Purified DNA concentrations were measured using the Qubit double-stranded DNA (dsDNA) BR assay kit (Thermo Fisher Scientific, USA), and samples were pooled in equimolar concentrations per library of 70 samples (randomly assigned to each library), concentrated using magnetic beads, and reeluted in 20 μL nuclease-free water. The final DNA concentration per library was measured using the Qubit system, after which the libraries were sent to GATC Biotech AG (Constance, Germany) (now part of Eurofins Genomics Germany GmbH) for 2× 150-bp sequencing on an Illumina HiSeq4000 instrument.

### Statistical analysis of larval performance and substrate properties.

All analyses were performed using R statistical software version 3.5.0 (69). Survival rates and percentages of prepupae were analyzed using generalized least-squares (GLS) regression, and individual larval weights were analyzed using generalized linear model (GLM) regression, using the gls (nlme) (70) and glm functions, respectively. Prior linear mixed-model (LMM) regression showed that a random term for batch (i.e., batch 1 or 2) was not needed for any performance parameter, based on Akaike’s information criterion (AIC) (71, 72). In the GLS regression, we selected a variance structure based on the AIC; a GLM with a gamma distribution was used if residuals violated GLS assumptions. The full model (substrate × density) was used as a fixed term. *Post hoc* comparisons were based on estimated marginal means (EMM) with Tukey-adjusted *P* values (emmeans package) (73).

The initial substrate pH was compared between substrates using an LMM with a random intercept for batch and an AIC-selected variance structure. The substrate moisture content on day 15 was analyzed in the same way as the larval performance parameters (GLS). The substrate pH on day 15 was analyzed with an LMM because a mixed model with a random term for batch fitted best based on the AIC. *Post hoc* comparisons for both the initial and final substrate pH were based on EMM with Tukey-adjusted *P* values.

### Bioinformatics and statistical analysis of microbiota data.

Raw amplicon sequence data were analyzed using the NG-Tax pipeline with default settings (32). In short, paired-end libraries were demultiplexed using read pairs with perfectly matching barcodes. ASVs were picked as follows: sequences were ordered by abundance per sample, and reads were considered valid when their cumulative abundance was ≥0.1%. Taxonomy was assigned using the SILVA database version 128 (74). ASVs are defined as individual sequence variants rather than a cluster of sequence variants with a shared similarity above a specified threshold such as operational taxonomic units. Data were analyzed using the phyloseq v1.24.2 (75) and microbiome v1.2.1 (76) packages. Chloroplast and mitochondrial 16S rRNA sequences were removed prior to analysis.

Contaminant ASVs were identified based on visual inspection of correlation plots between the DNA concentration and the relative abundance of each ASV, and these ASVs were removed from the data set prior to further analysis. Data quality was assessed by comparing the composition of the sequenced positive controls to the known composition (32) using Spearman’s rank correlation. Reproducibility was assessed by Spearman’s rank correlation of technical replicates (duplicate substrate samples within each substrate of one container with 0 or 200 larvae per container on days 5 and 15, and PCR duplicates [one substrate and two larval samples] across sequence libraries). Technical replicates with a Spearman correlation coefficient (*r*) of ≥0.7 were considered reproducible.

In the alpha (within-sample) diversity and constrained beta (between-sample) diversity analyses, we tested models separately on a data subset of substrates, including a density of 0 larvae per container, and on a data subset of larvae and substrates excluding this density and day 0 samples. This was done because parameter estimation and multivariate permutation tests required balanced data sets. This requirement did not apply to the unconstrained beta diversity analysis (nonmetric multidimensional scaling [NMDS]), so we included all data for that analysis. Data were not normalized to an equal sequencing depth because for data processed in NG-Tax, diversity does not depend on the sequencing depth (77).

The alpha diversity of the microbiota at the genus level was calculated as Faith’s phylogenetic diversity and Shannon diversity, using the picante and microbiome packages, respectively (78, 79), and tested for the significance of treatment effects using an LMM with a variance structure after AIC-based model selection.

Beta diversity at the genus level was visualized using NMDS (minimum of 100 iterations) (80), based on weighted UniFrac distances (81). The relative importance of substrate, larval density, and time in explaining microbiota composition was determined by distance-based redundancy analysis (dbRDA) directly decomposing the weighted UniFrac distances (dbrda function of the vegan package v2.5-6) (82–84). We compared the full model (substrate × density × time point) with a null model, and the significance of the main effects and interaction terms was tested using permutational multivariate analysis of variance (999 permutations) stratified for container identifier to account for repeated measures (anova.cca function of the vegan package) (85).

To assess the effect of larval density on the substrate microbiota within each substrate over time, we performed weighted UniFrac distance-based principal-response curves (dbPRCs), with the control without larvae as a baseline (function prc in the vegan package) (84, 86). In addition, the PRC returns an output on indicator taxa by means of species weights, indicating an association with treatments along the first PRC axis. Within each time point, we tested the effect of larval density on substrate microbiota composition and pairwise compared the axis scores of the first principal coordinate between larval densities (analysis of variance with Tukey contrasts). The same analysis was done on the larval microbiota alone (with a baseline at 50 larvae per container) to assess the effect of larval density on the internal microbiota, and within each feed substrate × density combination (with substrate as the baseline) to assess the difference between the larval and substrate microbiota over time.

### Data availability.

The sequence data sets generated and analyzed during the current study have been deposited in the European Nucleotide Archive (ENA) repository under the study accession number PRJEB40667 (87). The metadata for the 16S rRNA samples, the data on larval performance and pH, and the R script used for analyzing the data sets are available at the 4TU.ResearchData Repository (88).
