# Supplementary material for: Black Soldier Fly Larvae Influence Internal and Substrate Bacterial Community Composition Depending on Substrate Type and Larval Density
Source: Appl Environ Microbiol. 2022 May 9;88(10):e00084-22. doi: 10.1128/aem.00084-22 (PMC9128521; doi:10.1128/aem.00084-22)
Supplement: Supplemental file 1 — Text S1, Tables S1 to S6, and Fig. S1 to S6. Download aem.00084-22-s0001.pdf, PDF file, 1.5 MB [file aem.00084-22-s0001.pdf]

Supplemental material belonging to: "Black soldier fly larvae influence internal and substrate bacterial community composition depending on substrate type and larval density"

Stijn J.J. Schreven,[a]# Hugo de Vries,[b] Gerben D.A. Hermes,[b]* Giacomo Zeni,[a,b,$] Hauke Smidt,[b] Marcel Dicke,[a] Joop J.A. van Loon [a]

[a] Laboratory of Entomology, Plant Sciences Group, Wageningen University & Research, P.O. Box 16, 6700 AA, Wageningen, The Netherlands

[b] Laboratory of Microbiology, Agrotechnology & Food Sciences Group, Wageningen University & Research, P.O. Box 8033, 6700 EH, Wageningen, The Netherlands

# Address correspondence to Stijn Schreven, stijn.schreven@gmail.com

* Present address: Chr. Hansen A/S, Hørsholm, Denmark

[$] Present address: BugsLife s.r.l., Bevagna, Italy

Supplemental Text S1. Discussion on possible biofilms on the substrate surfaces

The layers of microbial biomass on the surface were avoided for substrate sampling because they likely contained a higher population density of bacteria and different community composition than the underlying substrate in which the larvae were found to be feeding in the later larval instars. Including these layers could have biased the bacterial community composition of the total substrate sample towards that of the surface layer. We judged that less relevant because the larvae mainly forage below this layer. The surface layers on camelina and chicken manure could have reduced the effects of time and larval density on substrate microbiota and the difference between larval and substrate microbiota, because they contained less *Lactobacillus,* and more *Serratia* and/or *Acinetobacter* (camelina) or more *Koukoulia* (chicken manure). Besides, this could have increased the effect of substrate type on microbiota, because *Lactobacillus* is shared across all three substrates, whereas *Koukoulia* is unique to chicken manure, and *Serratia* and *Acinetobacter* were already more abundant in camelina than in the other two substrates.

Table S1. Relative abundance of bacterial genera detected in no-template controls (NTC) of PCR.

**Genus**	**NTC 1**	**NTC 2**	**NTC 3**	**NTC 4**	**NTC 5**
Nesterenkonia	0.276	0.326	0.430	0.420	0.357
Caldalkalibacillus	0.126	0.113	0.348	0.310	0.329
Ralstonia	0.191	0.130	0.055	0.081	0.069
Halomonas	0.132	0.133	0.039	0.056	0.079
Bacillus	0.075	0.061	0.052	0.045	0.060
Bacillaceae (unassigned)	0.073	0.058	0.037	0.031	0.032
Shewanella	0.035	0.067	0.021	0.031	0.033
Halomonadaceae (unassigned)	0.025	0.040	0.012	0.020	0.021
Xanthomonadaceae (unassigned)	0.028	0.029	0	0	0.004
Cupriavidus	0.012	0.013	0.004	0.002	0.009
Achromobacter	0.005	0.004	0	0.002	0.002
Dietzia	0.005	0.005	0	0	0
Geobacillus	0.002	0.007	0	0	0
Georgenia	0.003	0.003	0	0	0
Unassigned taxa	0	0	0.002	0	0.003
Delftia	0	0.002	0	0.001	0.001
Rhodococcus	0.002	0.002	0	0	0
Sphingomonas	0.002	0.002	0	0	0
Aerococcus	0.003	0	0	0	0
Mycobacterium	0.003	0	0	0	0
Lactobacillus	0	0	0	0	0.002
Dermacoccus	0	0.001	0	0	0
Staphylococcus	0	0.001	0	0	0
Rubrobacter	0.001	0	0	0	0
Tetragenococcus	0	0.001	0	0	0
Comamonadaceae (unassigned)	0.001	0	0	0	0
Glutamicibacter	0.001	0	0	0	0
Brevundimonas	0	0.001	0	0	0

Ranked by relative abundance. Each NTC is from a different sequencing library.

Table S2. Total relative abundance (of all reads in dataset) of ASVs identified as contaminants.

ASV code	Genus	number of reads	% of total reads
301590670	Bacillaceae (unassigned)	34651	0.0511%
301590674	*Bacillus*	17336	0.0256%
301590679	*Bacillus*	10775	0.0159%
3015901111	*Bacillus*	1730	0.0026%
301590601	*Caldalkalibacillus*	88233	0.1302%
301590672	*Caldalkalibacillus*	34727	0.0512%
3015901123	*Caldalkalibacillus*	1624	0.0024%
3015901572	*Caldalkalibacillus*	7893	0.0116%
3015901605	*Caldalkalibacillus*	638	0.0009%
3015901109	*Cupriavidus*	4138	0.0061%
3015901814	*Delftia*	146	0.0002%
3015901112	*Dietzia*	1530	0.0023%
3015901113	*Georgenia*	751	0.0011%
301590676	Halomonadaceae (unassigned)	17890	0.0264%
301590660	*Halomonas*	43649	0.0644%
301590669	*Halomonas*	29361	0.0433%
3015901115	*Halomonas*	1106	0.0016%
301590591	*Nesterenkonia*	240340	0.3546%
3015901108	*Nesterenkonia*	16887	0.0249%
3015901126	*Nesterenkonia*	795	0.0012%
3015901798	*Nesterenkonia*	474	0.0007%
301590589	*Ralstonia*	123086	0.1816%
301590677	*Shewanella*	17149	0.0253%
301590741	*Shewanella*	7292	0.0108%
3015901110	*Shewanella*	2096	0.0031%
301590675	Xanthomonadaceae (unassigned)	11494	0.0170%
**Total**		**715791**	**1.0561%**

Contaminant ASV identification based on assessment of correlation plots between ASV relative abundance and DNA concentration of samples.

Table S3. Substrate microbiota variation of the three feed substates combined, partitioned by model terms (PERMANOVA of weighted UniFrac distances).

By terms	df	SSq	F	*p*	%
Substrate	2	8.49	82.35	0.001	38.2%
Density	3	0.99	6.37	0.003	4.4%
Timepoint	2	1.44	13.97	0.001	6.5%
Substrate:Density	6	1.16	3.75	0.016	5.2%
Substrate:Timepoint	4	2.45	11.87	0.001	11.0%
Density:Timepoint	6	0.64	2.07	0.001	2.9%
Substrate:Density:Timepoint	12	1.48	2.40	0.001	6.7%
Residual	108	5.56	NA	NA	

This analysis excludes substrate samples of day 0, because balanced datasets are required for restricted permutations. R^2 = 75%, *i.e.* the percentage of total microbiota variation explained by model Substrate x Density x Timepoint (weighted UniFrac distance-based redundancy analysis). Permutation test against null model (999 permutations): χ^2 = 16.64, F = 9.23, p = 0.001. Df = degrees of freedom, SSq = sum of squares, % = the percentage of microbiota variation explained by each model term, calculated as the relative sum of squares (sum of squares divided by total sum of squares).

Table S4. Substrate microbiota variation within each feed substrate, partitioned by model terms (PERMANOVA of weighted UniFrac distances).

	**Chicken feed**					**Camelina**					**Chicken manure**				
R^2	79%					48%					84%				
Test against null model	χ^2	10.01				χ^2	2.20				χ^2	5.73			
	F	11.75				F	3.07				F	16.46			
	p	0.001				*p*	0.001				*p*	0.001			
**By terms**	**df**	**SSq**	**F**	***p***	**%**	**df**	**SSq**	**F**	***p***	**%**	**df**	**SSq**	**F**	***p***	**%**
Density	3	0.57	3.33	0.001	4.5%	3	0.67	3.45	0.002	14.9%	3	0.70	10.16	0.001	10.3%
Timepoint	3	8.57	50.31	0.001	67.3%	2	0.55	4.19	0.001	12.0%	3	4.22	61.69	0.001	62.8%
Density:Timepoint	9	0.87	1.70	0.147	6.8%	6	0.98	2.50	0.003	21.5%	9	0.71	3.48	0.001	10.6%
Residual	48	2.73	NA	NA		36	2.35	NA	NA		48	1.10	NA	NA	

This analysis includes substrate samples of day 0 for chicken feed and chicken manure. R^2 is the percentage of total microbiota variation explained by the model Density x Timepoint (weighted UniFrac distance-based redundancy analysis). Df = degrees of freedom, SSq = sum of squares, % = the percentage of microbiota variation explained by each model term, calculated as the relative sum of squares (sum of squares divided by total sum of squares).

Table S5. Larval microbiota variation of the three substrates partitioned by model terms (PERMANOVA of weighted UniFrac distances).

**By terms**	**df**	**SSq**	**F**	***p***	**%**
Substrate	2	6.40	47.73	0.001	34.7%
Density	2	0.18	1.36	0.487	1.0%
Timepoint	2	1.24	9.25	0.001	6.7%
Substrate:Density	4	0.44	1.66	0.369	2.4%
Substrate:Timepoint	4	3.40	12.68	0.001	18.4%
Density:Timepoint	4	0.48	1.79	0.001	2.6%
Substrate:Density:Timepoint	8	0.89	1.65	0.001	4.8%
Residual	81	5.43	NA	NA	

R^2 = 71%, *i.e.* the percentage of total microbiota variation explained by the model Substrate x Density x Timepoint (weighted UniFrac distance-based redundancy analysis). Permutation test against null model (999 permutations): χ^2 = 13.04, F = 7.48, *p* = 0.001. Df = degrees of freedom, SSq = sum of squares, % = the percentage of microbiota variation explained by each model term, calculated as the relative sum of squares (sum of squares divided by total sum of squares).

Table S6. Larval microbiota variation within each feed substrate, partitioned by model terms (PERMANOVA of the weighted UniFrac distances).

	**Chicken feed**					**Camelina**					**Chicken manure**				
R^2	61%					46%					59%				
Test against null model	χ^2	2.43				χ^2	1.92				χ^2	2.29			
	F	5.17				F	2.86				F	4.87			
	p	0.001				*p*	0.002				*p*	0.001			
**By terms**	**df**	**SSq**	**F**	***p***	**%**	**df**	**SSq**	**F**	***p***	**%**	**df**	**SSq**	**F**	***p***	**%**
Density	2	0.26	2.22	0.001	6.5%	2	0.22	1.31	0.214	5.3%	2	0.15	1.25	0.057	3.8%
Timepoint	2	1.49	12.66	0.001	37.1%	2	1.47	8.81	0.001	35.3%	2	1.68	14.30	0.001	43.4%
Density:Timepoint	4	0.68	2.90	0.003	17.0%	4	0.22	0.66	0.72	5.3%	4	0.46	1.97	0.066	11.9%
Residual	27	1.59	NA	NA		27	2.26	NA	NA		27	1.58	NA	NA	

R^2 is the percentage of total microbiota variation explained by the model Density x Timepoint (weighted UniFrac distance-based redundancy analysis). Df = degrees of freedom, SSq = sum of squares, % = the percentage of microbiota variation explained by each model term, calculated as the relative sum of squares (sum of squares divided by total sum of squares).

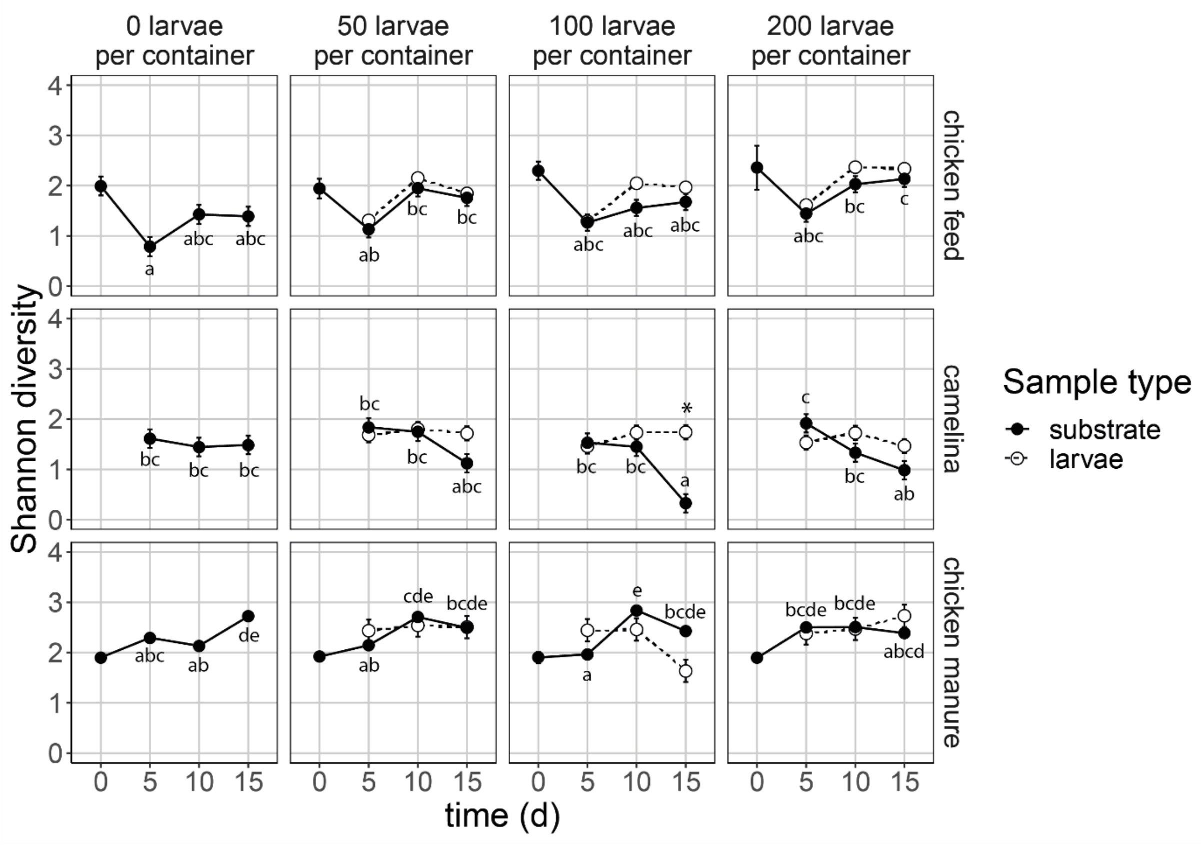

Fig. S1. Shannon diversity (estimated marginal mean ± SE, n = 4) of larval and substrate microbiota over time, in chicken feed (top row), camelina (middle), and chicken manure (bottom), separated by larval density. Within a diet, means of substrate microbiota diversity with no shared letters are significantly different (α = 0.05, Tukey contrasts from LMM regression on substrate samples, excluding day 0); means of larval microbiota diversity with an asterisk are significantly different from the corresponding substrate microbiota (α = 0.05, Tukey contrasts from LMM regression on substrate and larval samples, excluding samples of day 0 or 0 larvae per container).

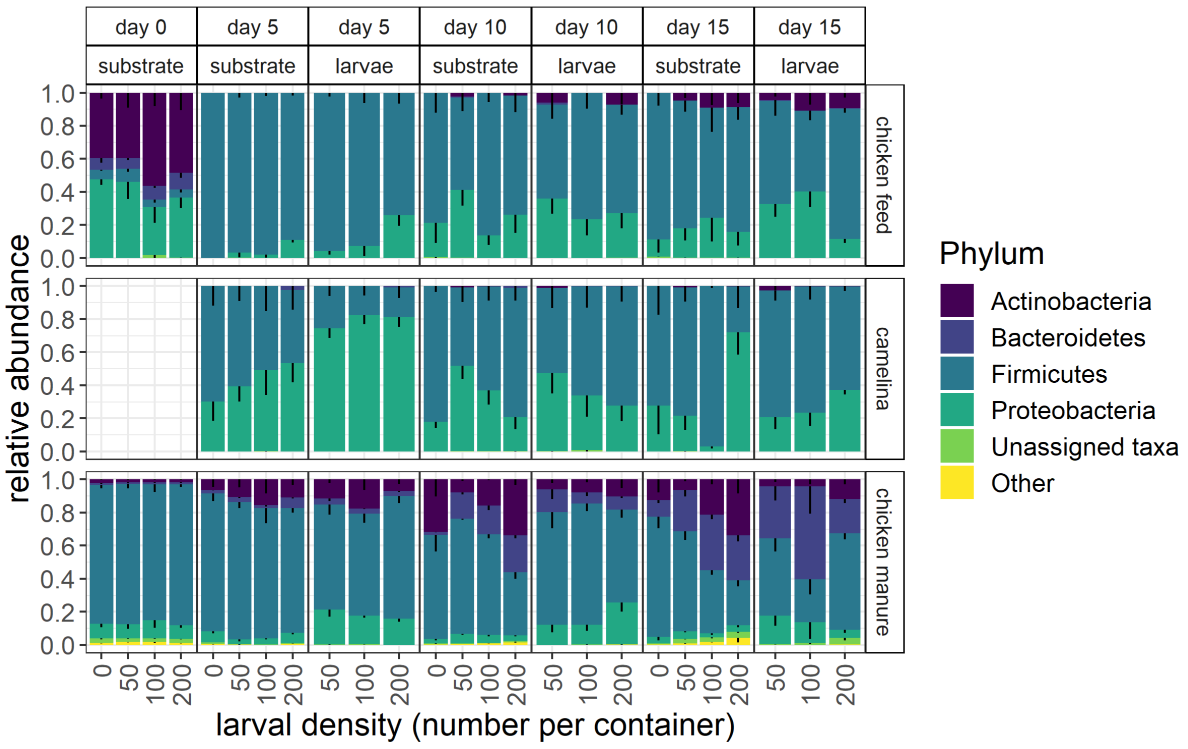

Fig. S2. Relative abundance of the five most abundant bacterial phyla in substrate and larval microbiota (mean - SE, n = 4).

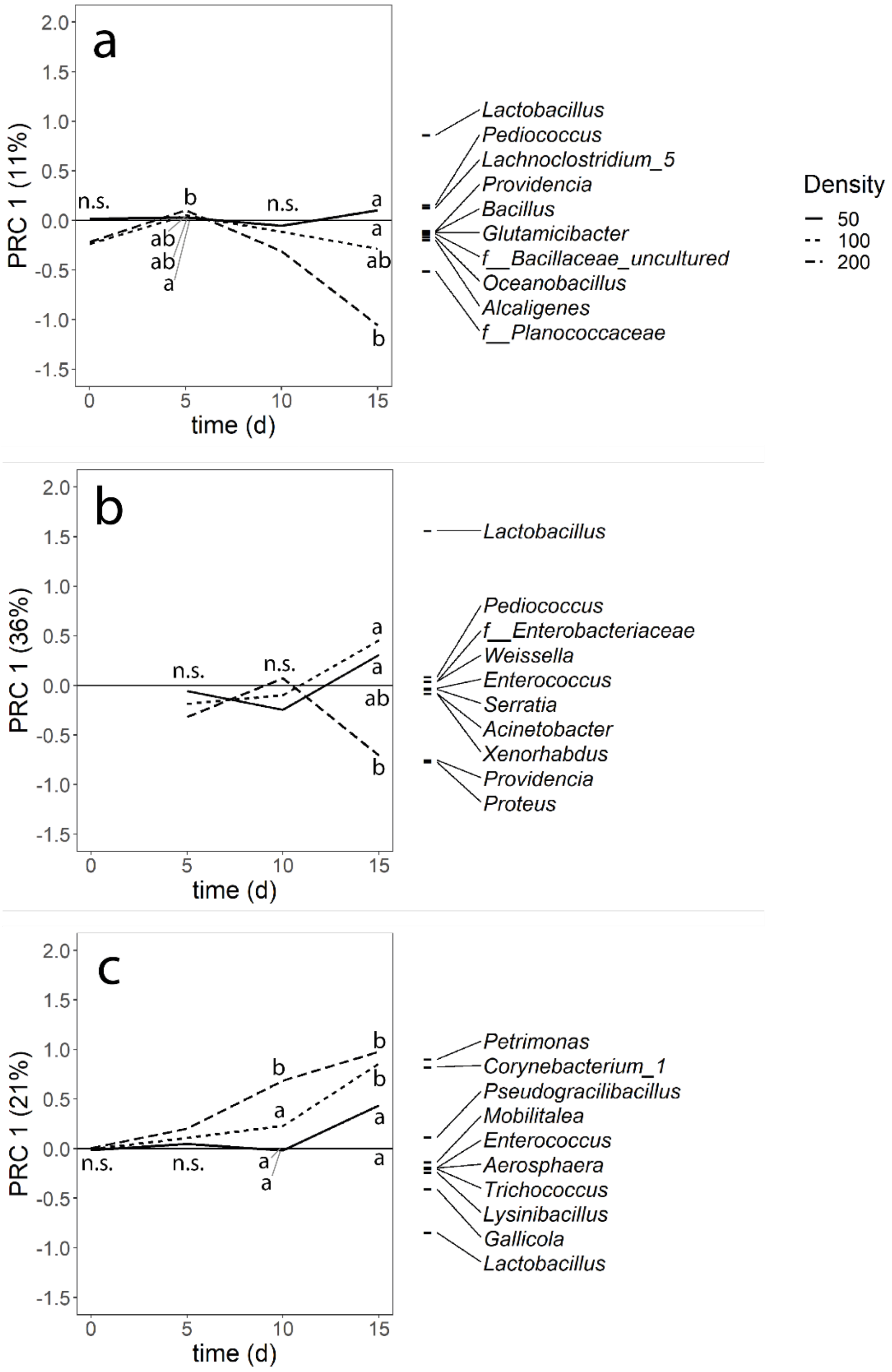

Fig. S3. Weighted UniFrac distance-based Principal Response Curves for substrate microbiota of different larval densities per feed substrate: (a) chicken feed, (b) camelina, (c) chicken manure. Baseline is the control treatment without larvae. The y-axis represents the canonical coefficient, *i.e.* the deviation in microbiota composition of a treatment group compared with the control group (baseline y = 0), limited to the part of total microbiota variation explained by the treatment, *i.e.* larval density (percentage in parentheses). Points that share no letters are significantly different on that day ($\alpha = 0.05$, Tukey contrasts between densities, on 1st axis scores per timepoint). At the right-hand margin of each plot, the genera with the top 10 absolute weights along the 1st PRC axis are displayed. The more positive the weight of a genus, the more likely the response of that genus follows the response curve of the community – high negative weights show the opposite pattern.

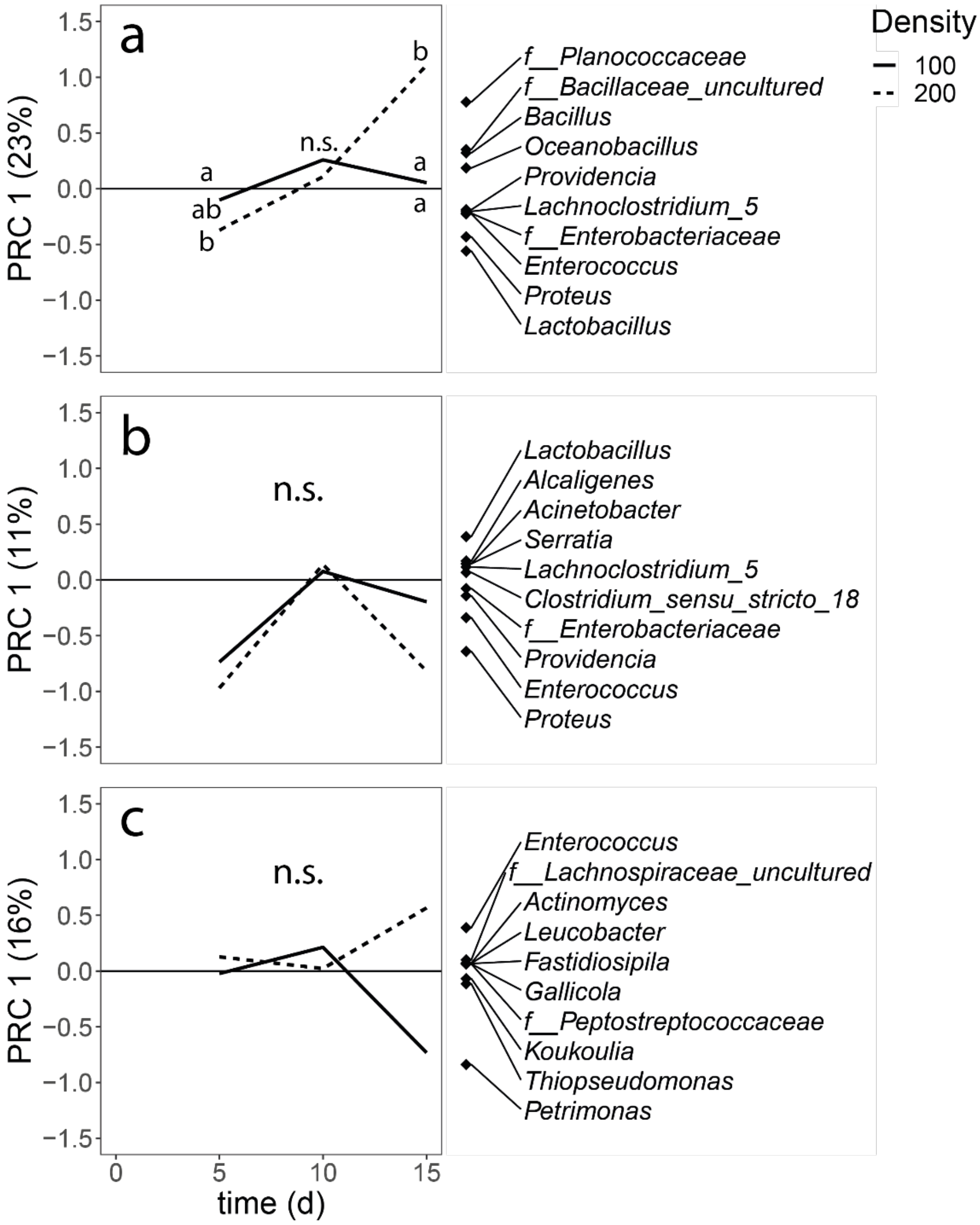

Fig. S4. Weighted UniFrac distance-based Principal Response Curves for larval microbiota of different larval densities per feed substrate: (a) chicken feed, (b) camelina, (c) chicken manure. Baseline is the treatment of 50 larvae per container. The y-axis represents the canonical coefficient, *i.e.* the deviation in microbiota composition of a treatment group compared with the control group (baseline y = 0), limited to the part of total microbiota variation explained by the treatment, *i.e.* larval density (percentage in parentheses). Points that share no letters are significantly different on that day ($\alpha = 0.05$, Tukey contrasts between densities, on 1st axis scores per timepoint). At the right-hand margin of each plot, the genera with the top 10 absolute weights along the 1st PRC axis are displayed. The more positive the weight of a genus, the more likely the response of that genus follows the response curve of the community – high negative weights show the opposite pattern.

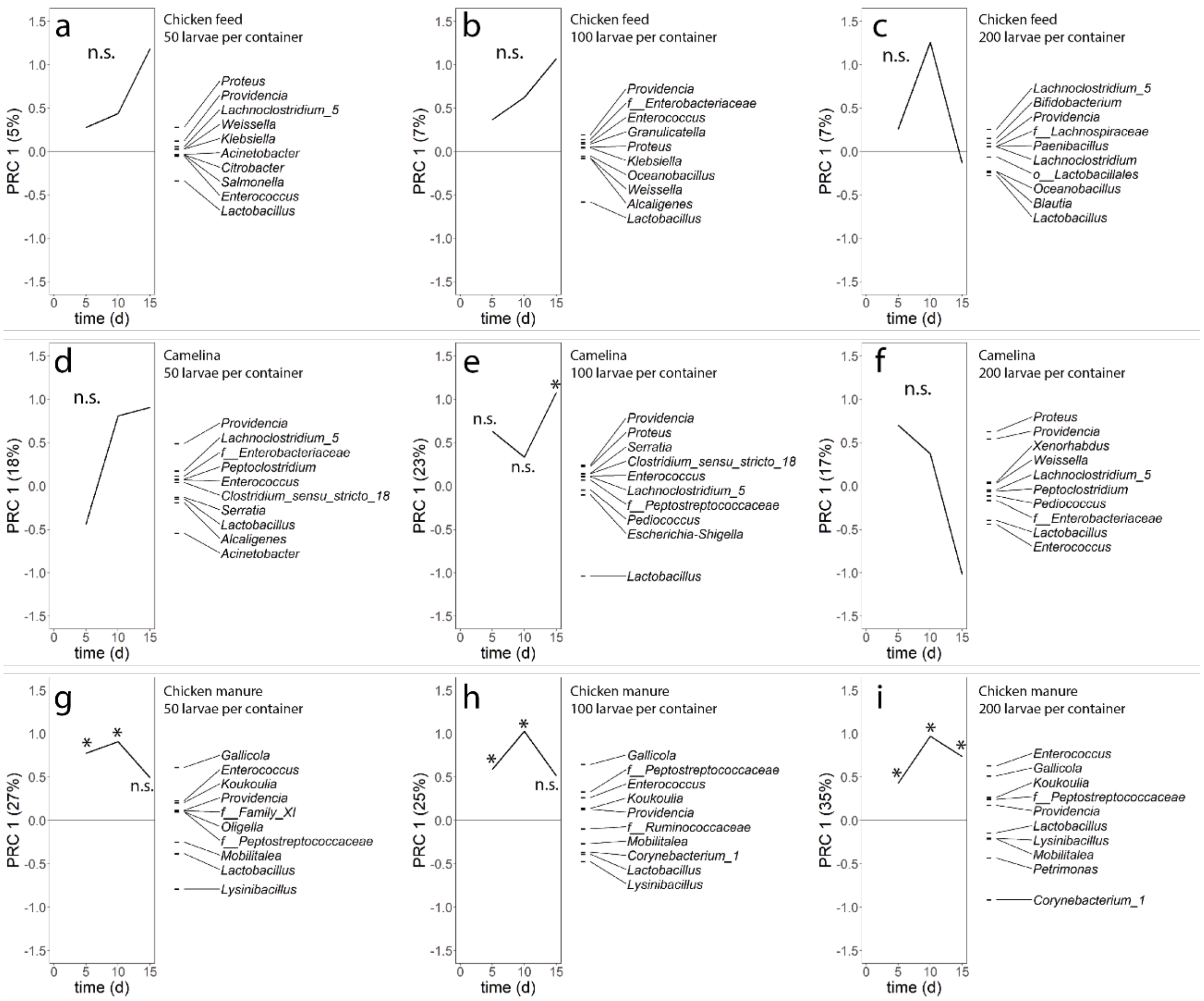

Fig. S5. Weighted UniFrac distance-based Principal Response Curves for larval and substrate microbiota per feed substrate and per larval density. (a-c) chicken feed, (d-f) camelina, and (g-i) chicken manure. Larval densities: (a, d, g): 50 larvae per container; (b, e, h): 100 larvae per container; (c, f, i): 200 larvae per container. Baseline is the substrate microbiota. The y-axis represents the canonical coefficient, *i.e.* the deviation in microbiota composition of a treatment group compared with the control group (baseline y = 0), limited to the part of total microbiota variation explained by the treatment, *i.e.* sample type (larvae or substrate; percentage in parentheses). Points with an asterisk indicate significant difference between larval and substrate microbiota on that day (α = 0.05, PERMANOVA test of distance-based Principal Response Curves for sample type). At the right-hand margin of each plot, the genera with the top 10 absolute weights along the 1st PRC axis are displayed. The more positive the weight of a genus, the more likely the response of that genus follows the response curve of the community – high negative weights show the opposite pattern.

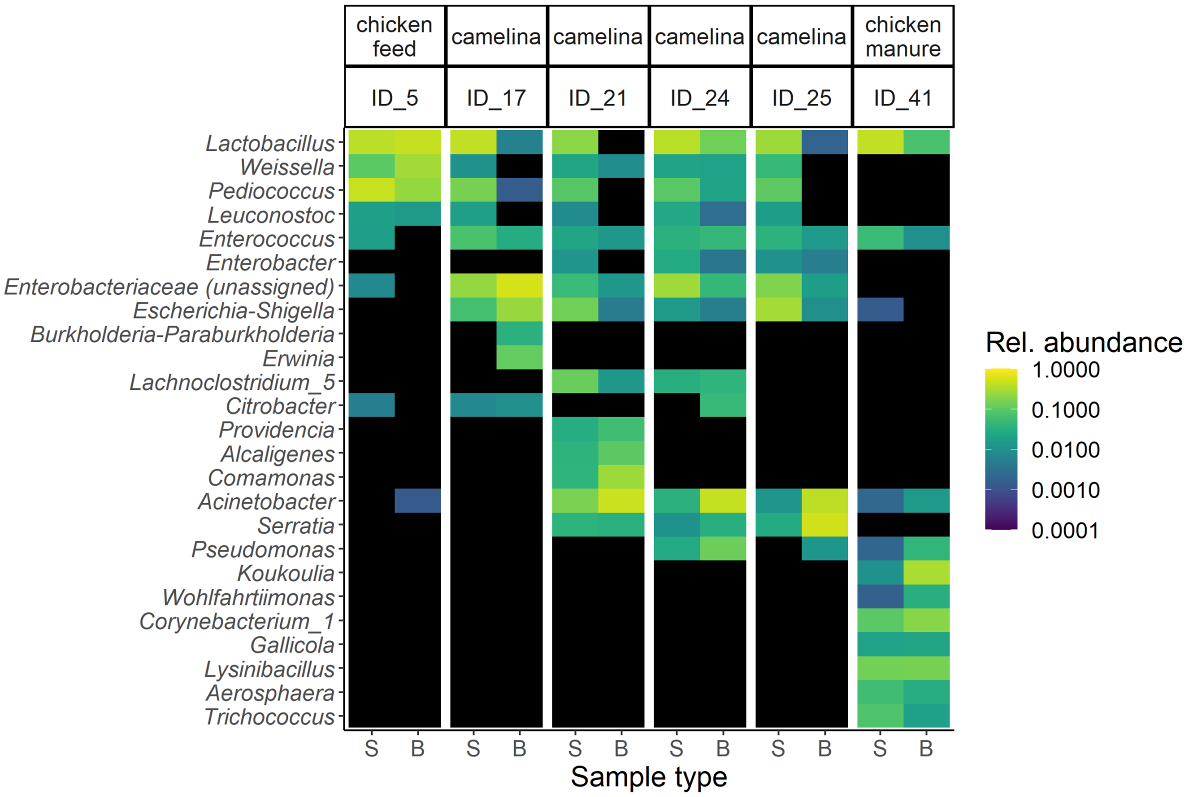

Fig. S6. Heatmap of relative abundance of the 25 most abundant bacterial genera in substrates (sample type "S") and biofilms (sample type "B") on day 5. ID = container ID.
